# Prescription Drug Monitoring Programs: Examining Limitations and Future Approaches

**DOI:** 10.5811/westjem.2014.10.24197

**Published:** 2015-01-06

**Authors:** Christopher A. Griggs, Scott G. Weiner, James A. Feldman

**Affiliations:** * Carolinas Medical Center, Department of Emergency Medicine, Charlotte, North Carolina; † Brigham and Women’s Hospital, Department of Emergency Medicine, Boston, Massachusetts; ‡ Boston University School of Medicine, Boston Medical Center, Boston, Massachusetts

## Abstract

Prescription drug abuse is a leading cause of accidental death in the United States. Prescription drug monitoring programs (PDMPs) are a popular initiative among policy makers and a key tool to combat the prescription drug epidemic. This editorial discusses the limitations of PDMPs, future approaches needed to improve the effectiveness of PDMPs, and other approaches essential to curbing the rise of drug abuse and overdose.

## INTRODUCTION

Prescription drug abuse is a leading cause of accidental death in the United States. Local, state, and federal agencies have implemented several policies to address this epidemic, including drug take-back programs, prescriber education, pain clinic laws, and prescription drug monitoring programs (PDMP). PDMPs are a popular initiative among policy makers, as they easily provide clinicians with scheduled medication histories, helping identify patients that may be diverting medications or abusing them. As of October 2014, 22 states have passed laws mandating that providers use the PDMP in certain circumstances. However, enthusiasm for PDMPs as a key tool to combat the prescription drug epidemic may cause proponents and policy makers to overlook their potential limitations. This enthusiasm may also prevent the development of more comprehensive and evidence-based strategies to address this public health crisis and the conclusion that additional steps are needed to combat the opioid epidemic.

Evidence to support the effectiveness of PDMPs comes largely from observational studies or surveys of providers.^1^ Recent data from Florida show a decline in prescription drug overdose deaths and doctor shopping after the implementation of their PDMP and pain clinic law.^2^,^3^ Virginia also reported a fall in the number of “doctor shoppers” after implementation.^4^ Additionally, national data from the Centers for Disease Control (CDC) show that overdose deaths due to opioid analgesics decreased by 5% from 2011 to 2012, the first decrease in a decade.^5^ It is not clear if PDMPs were responsible for this decline or if other interventions, such as laws limiting dispensing of medications from pain clinics and overall prescriber awareness of the risks of opioids, led to this decline. Contrary to this evidence, previous studies examining PDMP effects on opioid prescribing show mixed effects before 2008, with some states having reduction in prescribing and overdose deaths and others showing an increase.^6^–^8^ While PDMPs are likely contributing to the overall decline in drug diversion and prescription opioid overdoses, the true effect of PDMPs is to be determined and there are several substantial limitations that should be addressed.

### PDMP Data – Devil in the Details

PDMPs identify “doctor shopping” through unsolicited reports sent from government agencies to clinicians, surveillance of aberrant prescribing behavior to identify irresponsible prescribing and by clinician review of patient reports before prescribing. PDMP databases generate data from pharmacies directly reporting to the state when a prescription is filled. States have varying delays in how long it takes for the data to appears in the database, for example in Massachusetts, there is up to a three-week delay. For data to be accurate, the name and date of birth must be reported correctly by the patient, written correctly on the prescription, entered correctly by the pharmacy, and again entered correctly by the clinician searching for the report. Any error may generate an incorrect report. Currently only 22 states require a patient to show identification before dispensing a controlled substance, allowing “doctors shoppers” and “pill mills” to easily deceive the system.^9^ Improved identification at both the point of prescribing and dispensing should be explored as a means to improve the effectiveness of PDMPs.

The PDMP relies on Drug Enforcement Administration (DEA) numbers to identify prescribers. In the case of residents and moonlighting clinicians, many hospitals use hospital-based DEA numbers and the database reports the hospital name instead of the specific prescriber. If a patient sees multiple providers at the same clinic, the database is unable to indicate whether the providers are working together. Such a profile may lead a clinician to inappropriately conclude a patient is “doctor shopping,” when the patient is, in fact, following up correctly. The confusion created by DEA numbers could be remedied if further information was provided on the PDMP database that indicated a prescriber’s specialty and association with a specific clinic or group. Additionally, hospital-based DEA numbers should be registered with the state PDMPs to give prescriber specific information.

To date, there is no agreed upon threshold to define questionable behavior, and each government agency or clinician is left to decide what criteria should cause them concern. The lack of objective criteria creates a challenge for clinicians who are balancing their duty to treat pain, to meet patient expectations, and to prevent misuse and diversion in their communities. The Massachusetts Department of Public Health recommends discussing concerning PDMP profiles with patients and to use the PDMP in the context of a complete patient evaluation, including review of outside medical records, and discussions with other providers.^10^ There is, however, no guidance on how to interpret the report in this context.

Recent studies have shown increases in mortality in patients with greater than four providers, greater than four pharmacies and using greater than 100 morphine milligram equivalents per day.^11^ However, using any absolute value results in identifying patients as “doctor shoppers” or at risk for overdose who, in fact, are not. Many patients have multiple prescribers because of poor primary care access, visits to emergency departments (ED) for acute exacerbations of pain, and conditions requiring visits to multiple specialists. Having to interpret the PDMP in this context allows bias and other factors outside of objective data to determine who is labeled as at risk or not.

### PDMP and Sources of Opioids

PDMP effectiveness is dependent on the amount of misuse and diversion that results from clinician prescribing. Studies examining the PDMP profiles of those who died from prescription drug overdoses report the percentage of deaths related to “doctor shopping” range from 21% to 32%.^12^ Among those using opioids for nonmedical purposes, a national survey identified that 20% of individuals received opioids from more than one prescriber, while the remaining received opioids from their friends, family, drug dealers, or strangers.^13^ It is unknown how much of diverted medications result from “doctor shopping.” Diversion may alternatively result from patients with one prescriber, theft, or falsified prescriptions. PDMPs are therefore unable to identify many important sources of diversion and interventions are needed to target the other causes of diversion.

### PDMP effects on prescribing

Clinical studies depict mixed effects of PDMP reports on prescribing. Baehren et al.^14^ found PDMP use changed emergency physicians’ prescription plans in 41% of cases and resulted in less prescribing. Another study by Weiner et al.^15^ found PDMP data influenced prescribing behavior in only 9.5% of cases and resulted in more prescribing. Baehren et al.^14^ enrolled 18 providers but four providers were responsible for 63% of the patient encounters, compared to the Weiner et al.^15^ study that enrolled 38 providers and limited the participation of any one provider to 10%. The true effect of PDMPs on prescribing is likely closer to the results in the Weiner et al. study due to the bias inherent in the Baehren et al. study; however, further investigation is needed.

### Where do we go from here?

PDMPs are a valuable tool in concept, but their effectiveness must still be proven. Patients determined to deceive the system may do so by crossing state borders in states without effective data sharing or reporting false personal information when registering with hospitals and clinics. It also remains unclear if patients chronically treated with opioids will be adversely affected by PDMPs. In particular, pain patients with fragmented care and a poor primary care network are more likely to have a suspicious PDMP profile and may be undertreated.

The promise of PDMPs is to improve data sharing among providers in order to avert diversion and prescribing to those at risk of abuse and overdose. However, this data sharing is limited to a few data points. PDMPs could provide means of communication between providers within the Internet portal that is compliant with privacy laws and allows better communication on opioid prescribing. This would also allow emergency providers to notify other prescribers of patients who have either overdosed, are at risk for overdose, or have a pain contract.

If PDMPs are to be successful, further improvements are needed to improve accuracy, accessibility and interpretability of the data. Easy access with little effort on the part of the clinician is essential to increased usage. Even with legal mandates, enforcement will be challenging and clinicians are already overloaded with work, making PDMP review for all patients a challenge in many clinical settings. Further funding to integrate PDMP data into medical records is essential. Effective use of the PDMP will require studies determining how the PDMP should be used alongside the complete clinical encounter and to identify what values in a PDMP report should trigger intervention from the clinician.

While PDMPs are one tool in the fight against the opioid epidemic, they are not the panacea and a more comprehensive approach is needed. Our profession must come to consensus on the indications for opioid pain medications and their appropriate use in managing acute and chronic pain. Training clinicians in chronic pain management and responsible opioid prescribing may do more to reduce opioid prescribing than access to PDMPs. Improved patient education for those receiving opioids is also needed so our patients fully understand the risks and benefits of opioid therapy.

The aforementioned CDC data show a decrease in opioid analgesic overdose in 2012, but also show a 35% increase in heroin deaths over the same year and a continued rise in drug overdose deaths overall.^4^ If current interventions are able to decrease abuse and overdose from prescription opioids, the overdose epidemic may rage on from opioids provided through the black market. It is not enough to simply refuse to prescribe opioids to those with a concerning PDMP profile, but physicians must have candid conversations with their patients, particularly in the ED. Adequate funding is needed for drug abuse treatment programs, which will allow ED referrals to be more effective. Additionally, overdose education and naloxone distribution has shown promise in reducing opioid overdose death.^16^ The ED is a particularly critical location where naloxone distribution could be effective and further research on ED distribution of naloxone is warranted.

We are at a critical point in the opioid epidemic, and the path forward requires addressing opioid addiction and abuse via multiple methods. PDMPs have shown promise but have limitations and we must work to improve their effectiveness. ED providers are essential to identifying and participating in these improvements and expanding the discussion on how to effectively prevent overdose and abuse of opioids.
